# Função Endotelial por Dilatação Mediada por Fluxo (FMD) na Artéria Braquial em Hipertensos

**DOI:** 10.36660/abc.20240533

**Published:** 2025-05-15

**Authors:** Elaine Alves Santos Tessier, Carla Daltro, Eduardo Martins Netto, Glauco Moniz de Aragão Doria, Andrea Jimena Gutierrez Peredo, Fabio Bulhões, Roque Aras, Ryan A. Harris

**Affiliations:** 1 Universidade Federal da Bahia Salvador BA Brasil Universidade Federal da Bahia, Salvador, BA – Brasil; 2 Augusta University Laboratory of Integraative and Vascular Exercise Physiology Augusta Georgia EUA Augusta University - Laboratory of Integraative and Vascular Exercise Physiology – LIVEP, Augusta, Georgia – EUA

**Keywords:** Artéria Braquial, Endotélio Vascular, Hipertensão

## Abstract

**Fundamento:**

Valores elevados de pressão arterial (PA) têm sido tradicionalmente associados ao risco de doença isquêmica do coração, acidente vascular cerebral, doença renal crônica e mortalidade precoce. A dilatação mediada por fluxo (FMD) da artéria braquial após a desinsuflação do manguito tornou-se o parâmetro padrão para quantificar a função endotelial, sendo um desfecho substituto útil em função de sua não invasividade, estreita correlação com a função endotelial coronariana e associação com a incidência de eventos coronarianos em longo prazo.

**Objetivos:**

Testar hipóteses de correlação entre a FMD e diversos parâmetros sanguíneos e comparar os parâmetros entre grupos com a FMD alterada e não alterada e entre hipertensos dos grupos hipertensão arterial resistente (HAR e não HAR).

**Métodos:**

Setenta e dois voluntários de um ambulatório de referência em Hipertensão Arterial foram incluídos neste estudo transversal prospectivo, no qual foram comparadas diversas variáveis mensuradas nos pacientes, entre os grupos FMD alterada (n = 38) e não alterada (n = 34), e também entre os grupos HAR (n = 49) e não HAR (n = 23). Investigamos também quais variáveis explicariam a FMD. As análises estatísticas foram conduzidas por meio de métodos paramétricos quando os pressupostos foram atendidos e não paramétricos quando estes não foram atendidos. O nível de significância adotado nas análises estatísticas foi de 5%.

**Resultados:**

Os resultados mostraram uma correlação positiva significativa entre FMD e LDL (p = 0,204, p = 0,042) e entre FMD e triglicerídeos (p = 0,247, p = 0,037). Hemoglobina glicada foi maior no grupo HAR (p = 0,020), potássio foi maior no grupo não HAR (p = 0,029) e proteína C reativa foi maior no grupo HAR (p = 0,04). Não houve diferenças estatísticas significativas para as demais comparações.

**Conclusões:**

O LDL e os triglicerídeos são preditores da FMD e os grupos HAR e não HAR diferem quanto à quantidade de potássio, proteína C e hemoglobina glicada. Os grupos de FMD alterada e não alterada diferem somente em relação aos triglicerídeos.

## Graphical abstract

**Figura Central:**
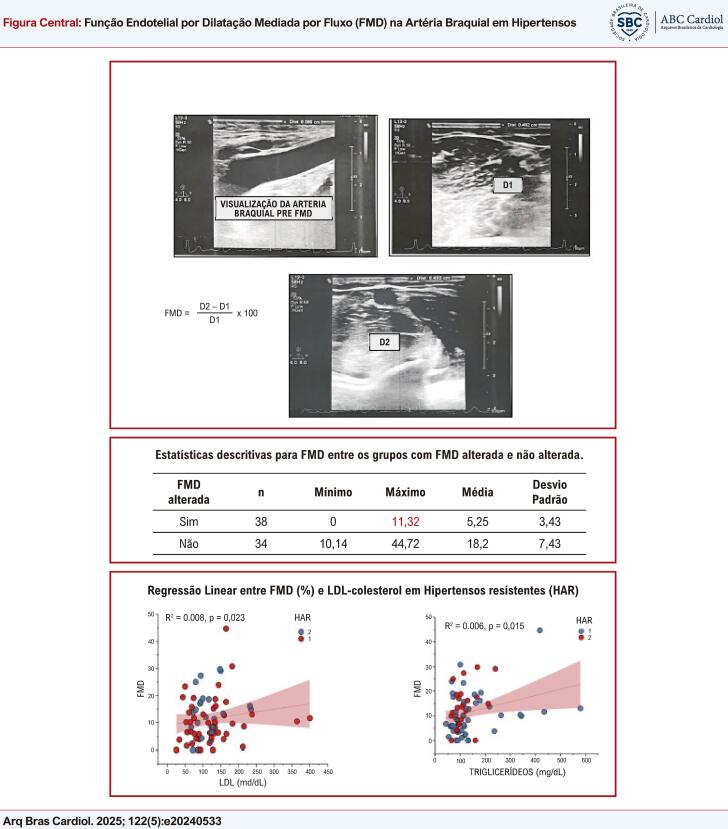
Função Endotelial por Dilatação Mediada por Fluxo (FMD) na Artéria Braquial em Hipertensos

## Introdução

Valores elevados de pressão arterial (PA) têm sido tradicionalmente associados ao risco de doença isquêmica do coração, acidente vascular cerebral, doença renal crônica e mortalidade precoce.^1^ A hipertensão arterial resistente (HAR) é caracterizada por PA de consultório, mantendo-se ≥ 140/90 mmHg mesmo com o uso de três ou mais medicamentos anti-hipertensivos com ações sinérgicas, em doses máximas preconizadas ou toleradas. Preferencialmente, pelo menos um desses medicamentos deve ser um diurético tiazídico. Quando o controle da PA é alcançado com o uso de quatro ou mais fármacos anti-hipertensivos, o paciente é considerado hipertenso resistente, porém controlado (PA < 140/90 mmHg).^1 - 4^ No Brasil, o estudo multicêntrico ReHOT (tratamento ideal para a hipertensão resistente) encontrou prevalência de 11,7% para a comorbidade em questão.^5^

A principal função do sistema arterial é “distribuir eficientemente o sangue aos órgãos periféricos e manter a homeostase vascular”.^3 , 6^ A disfunção endotelial é a primeira alteração funcional detectável no processo aterosclerótico.^7^ Isso se deve à diminuição da biodisponibilidade do óxido nítrico encontrada não apenas em pacientes que apresentam doença aterosclerótica clinicamente evidente, mas também em pacientes com fatores de risco.^8^ Trata-se de uma manifestação precoce tanto da doença aterosclerótica quanto do *diabetes mellitus* tipo 2 (DM2) e sua atenuação pode ocorrer logo após o início de terapias com efeitos antiateroscleróticos.^2 , 4^

Em 1992, Celermajer et al.^9^desenvolveram um método não invasivo, conhecido como dilatação mediada por fluxo (FMD), com o intuito de avaliar alterações precoces na função vascular em artérias sistêmicas. A FMD da artéria braquial após a desinsuflação do manguito tornou-se o parâmetro padrão para quantificar a função endotelial, sendo um desfecho substituto útil em função de sua não invasividade, estreita correlação com a função endotelial coronariana e associação com a incidência de eventos coronarianos em longo prazo.^7^ Há inúmeros potenciais fatores que podem confundir a mensuração da FMD.^10^Ademais, não há um consenso sobre aspectos como local e tempo de compressão do manguito, o que gera alterações no resultado quantitativo do exame.^11^

O objetivo do presente estudo é testar hipóteses de correlação entre a FMD e os diversos parâmetros sanguíneos e comparar os parâmetros entre grupos com FMD alterada e não alterada e entre hipertensos dos grupos HAR e não HAR.

## Métodos

### Coleta de dados

Setenta e dois voluntários de um ambulatório de referência em Hipertensão Arterial foram incluídos neste estudo transversal prospectivo. Os pacientes foram informados previamente sobre o preparo para a realização do exame.

As características clínicas e demográficas dos pacientes foram coletadas em prontuário eletrônico. O comitê de ética local aprovou o estudo (CAE n^o^. 81701717.6.0000.0049 e Parecer nº. 2.635.984) e o consentimento informado por escrito foi obtido de todos os pacientes antes da realização dos exames.

### Exame de dilatação mediada por fluxo da artéria braquial direita

Durante o exame, a vasodilatação na artéria braquial é avaliada e ocorre em resposta ao aumento significativo do fluxo sanguíneo, induzido por um período de oclusão circulatória. A hiperemia reativa é induzida pela liberação rápida de um manguito de pressão pneumático colocado ao redor do antebraço e insuflado até a pressão suprassistólica por cinco minutos. Esse procedimento é capaz de aumentar a tensão de cisalhamento exercida ao longo do vaso, de forma paralela e laminar, ativando mecanorreceptores nas células endoteliais e promovendo a liberação de óxido nítrico (NO).^12 - 14^

A pressão exercida no braço provoca isquemia vascular e consequente dilatação dos vasos.^15^ Atingidos os cinco minutos estabelecidos, a válvula de controle de deflação é aberta lentamente. Ao chegar a zero no aparelho, o diâmetro posterior (D2) é mensurado. Em seguida, aguarda-se um período de mais 60 segundos para aplicação da fórmula (D2 – D1)/D1 × 100, onde valores iguais ou inferiores a 10% estabelecem alterações, conforme estudo de Regattieri et al.^16^ Esse cálculo fornece os valores para o cálculo da FMD, objeto do estudo.

Todos os exames foram realizados pelo mesmo médico voluntário, especialista com 20 anos de atuação na área de ecoangiologia, e, naquela ocasião, oferecemos aos pacientes a oportunidade de também realizar o Doppler carotídeo e vertebral (c/v), obedecendo à metodologia descrita na Diretriz Brasileira de Dislipidemia 2017.^17^ Esse exame é sempre realizado antes da mensuração da FMD. Os marcadores laboratoriais utilizados para o estudo foram: Colesterol total, HDL - colesterol, LDL - colesterol, hemoglobina glicada (HbAc1), ácido úrico e proteína C reativa (PCR), coletados em prontuário eletrônico.^18^

### Análise estatística

Os dados foram compilados e analisados utilizando o software SPSS^®^ (versão 25.0, Chicago, IL Statistical Package for the Social Sciences). Além disso, utilizou-se um nível de significância alfa de 5% em todos os testes. As variáveis categóricas foram expressas em frequência e porcentagem, enquanto as variáveis contínuas foram expressas com média e desvio padrão. Para testar associações entre fatores categóricos, utilizou-se o teste qui-quadrado. Ao comparar o padrão de correlação entre variáveis quantitativas e ordinais, utilizou-se a análise de correlação de Spearman, que captura melhor os padrões de correlação positivos ou negativos sem a necessidade de linearidade perfeita. Ao comparar variáveis contínuas entre os grupos HAS (resistente e não resistente) e DMF (alterada e não alterada), utilizou-se o teste t para amostras independentes. Em todos os testes, a normalidade foi aferida pelo teste de Shapiro Wilk e a homogeneidade das variâncias, pelo teste de Levene. Os dados não se afastaram da normalidade, apresentando variâncias homogêneas em todas as comparações. Foram realizadas ainda análises de regressão linear entre LDL e FMD e entre Triglicerídeos e FMD, com os pressupostos de homocedasticidade e normalidade dos resíduos testados.

## Resultados

Para uma representação objetiva, a figura central ilustra os dados de maneira elucidativa e prática.

**Figura 1:**
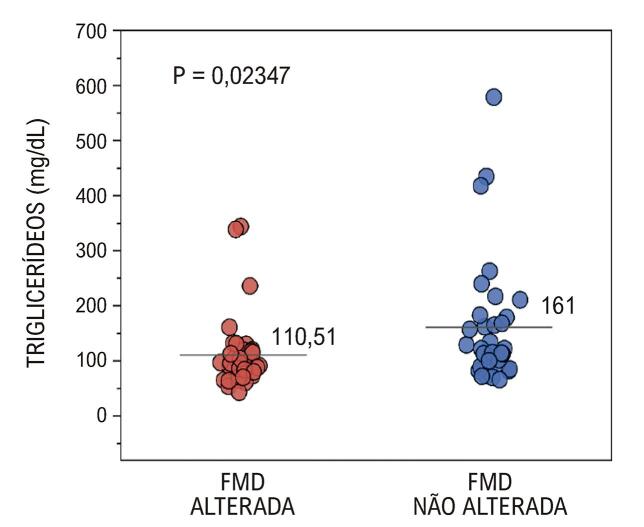
– Teste t comparando quantidade de triglicerídeos entre os grupos com FMD alterada e não alterada. FMD: dilatação mediada por fluxo.

Inicialmente, foram comparadas diversas variáveis entre os grupos com FMD alterada e não alterada ( Tabela 1 ). Pode-se notar diferenças significativas em relação aos triglicerídeos (p = 0,023), cujos níveis foram maiores no grupo com FMD não alterada ( Figura 1 ).

**Tabela 1:** – Perfil sociodemográfico e clínico dos hipertensos submetidos à Dilatação de Fluxo Mediado (FMD) na artéria braquial direita

Variável	FMD alterada (n = 38)	FMD não alterada (n = 34)	p
Idade (anos)	59,13 (10,8)	58,24 (13,02)	0,750
IMC (kg/m^2^)	30,9 (5,56)	30,67 (5,03)	0,855
PAS antes da FMD (mmHg)	143,39 (20,78)	144,32 (23,32)	0,859
PAD antes da FMD (mmHg)	85 (14,34)	84,29 (12,4)	0,824
Glicemia em jejum (mg/dL)	109,24 (31,53)	114,24 (43,81)	0,581
HbA1c (mg/dL)	6,31 (1,01)	6,39 (1,05)	0,728
Colesterol total (mg/dL)	188,37 (64,28)	185 (51,73)	0,809
Triglicerídeos (mg/dL)	110,51 (65,48)	161 (113,62)	0,023
HDL – colesterol (mg/dL)	49,01 (12,42)	47,03 (8,84)	0,445
LDL – colesterol (mg/dL)	116,43 (60,25)	127,63 (71,13)	0,475
Sódio (mg/dL)	139,74 (4,23)	140,78 (3,41)	0,265
Potássio (mg/dL)	4,34 (0,5)	4,29 (0,52)	0,667
Ácido úrico (mg/dL)	5,69 (1,49)	5,71 (1,76)	0,949
Proteína C reativa (mg/dL)	4,87 (4,06)	4,1 (3,43)	0,400
Frequência de consumo de bebida alcoólica	0,55 (1,06)	0,53 (1,26)	0,932
Frequência de prática de atividade física	1,53 (2,19)	2,06 (2,82)	0,371

Fonte: autoria própria. Nas células média e desvio padrão, o p estatístico foi obtido via teste t para amostras independentes. FMD: dilatação mediada por fluxo; IMC: índice de massa corporal; PAS: pressão arterial sistólica; PAD: pressão arterial diastólica.

Na comparação entre os grupos de pressão sanguínea, foi encontrado um valor de mediana maior para o grupo não HAR (43,9) em comparação com o grupo HAR (33), p = 0,039. Além disso, foram testadas as correlações entre FMD e todas as variáveis quantitativas do estudo ( Tabela 2 ).

**Tabela 2:** – Correlação de Spearman entre FMD e as demais variáveis quantitativas

Variáveis	N	Spearman (Rho)	p
Idade	72	-0,004	0,972
IMC	72	0,023	0,844
PAS antes da FMD	72	-0,005	0,969
PAD antes da FMD	72	-0,062	0,602
glicemia de jejum	71	0,015	0,901
HbAc1	71	0,024	0,845
Colesterol total	72	0,122	0,306
HDL – colesterol	71	0,034	0,776
LDL – colesterol	71	0,204	0,042
Triglicérides	71	0,247	0,037
Sódio	70	-0,005	0,965
Potássio	70	0,022	0,859
Ácido úrico	69	-0,028	0,820
Proteína C-reativa	70	-0,111	0,360
Consumo de bebida alcoólica	72	-0,100	0,402
Prática de atividade física	72	0,106	0,374

Ao se analisar a estatística descritiva da nossa amostra, pode-se notar que o valor máximo obtido no grupo com FMD alterada foi de 11,32, com média de 5,25, desvio padrão de 3,43 e o valor mínimo foi zero. A FMD inalterada apresenta variação muito maior, com média de 18,2 e desvio padrão de 7,43, com valor mínimo de 10,14 e valor máximo de 44,72 (conforme Tabela 3 ). Ao considerar simplesmente o valor máximo do grupo da FMD como ponto de corte, esse valor seria de 11,32. No entanto, recomendam-se estudos elaborados especificamente para a questão do ponto de corte a fim de esclarecer melhor o problema.

**Tabela 3:** – Valores máximos e mínimos da FMD da artéria braquial direita em pacientes hipertensos resistentes

FMD alterada	N	Mínimo	Máximo	Média	Desvio Padrão
Sim	38	0	11,32	5,25	3,43
Não	34	10,14	44,72	18,2	7,43

FMD: dilatação mediada por fluxo

Observou-se uma correlação significativa positiva entre FMD e LDL e entre FMD e triglicerídeos. Foram realizadas análises de regressão para as duas relações significativas acima (Figuras 1 e 2).

**Figura 2:**
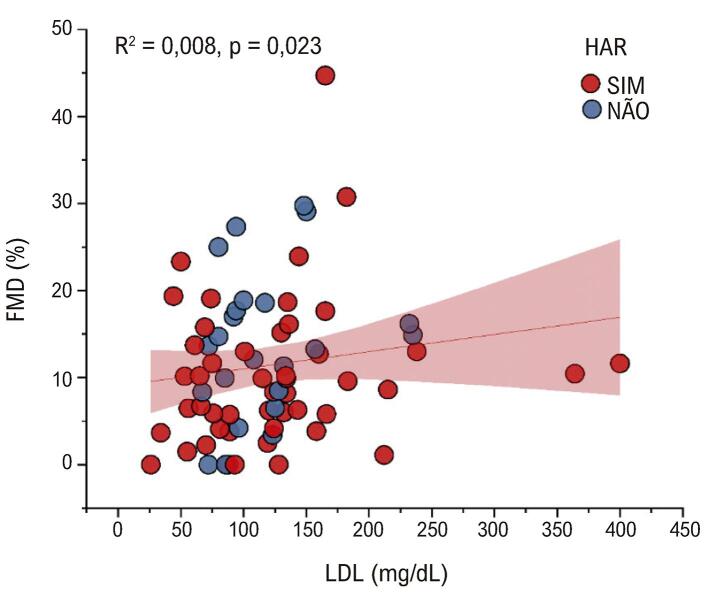
– Regressão linear entre FMD e LDL. FMD: dilatação mediada por fluxo; HAR: hipertensão arterial resistente.

Quanto maiores os valores do LDL, maiores os valores da FMD. O LDL prevê e explica corretamente 0,8% da variação da FMD ( Figura 2 ).

A Tabela 4 mostra uma associação entre os fatores categóricos e os grupos HAR e não HAR.

**Tabela 4:** – Pacientes hipertensos resistentes e não resistentes e variáveis categóricas

	Categoria	HAR	Não HAR	p
FDM alterada	Sim	29	9	
	Não	20	14	0,112
Tabagismo	Sim	2	1	
	Não	47	22	0,958
Consumo de bebida alcoólica	Sim	2	0	
	Não	35	20	0,299
Prática de atividade física	Sim	21	9	
	Não	28	14	0,765
IMC	Eutrófico	6	5	
	Sobrepeso	16	8	
	Obeso	27	10	0,508
Disglicêmico	Sim	29	14	
	Não	20	9	0,892
Vasodilatador	Sim	17	1	
	Não	32	22	0,006
Ateromatose	Sim	36	16	
	Não	13	7	0,73
	Não	13	12	0,033
Estatina	Sim	38	16	
	Não	11	7	0,466

Associações inferidas via teste de qui-quadrado. FMD: dilatação mediada por fluxo; IMC: índice de massa corporal.

O uso de vasodilatadores está mais associado a pacientes com HAR (0,006). A comparação das médias de LDL entre os grupos que usam e não usam estatina não mostrou diferenças significativas (p = 0,336). A Tabela 5 apresenta comparações de diversos parâmetros entre os grupos HAR e não HAR.

**Tabela 5:** – Hipertensos resistentes e não resistentes x variáveis quantitativas

Variável	HAR	não HAR	p
Idade	59,41 (11,4)	57,22 (12,7)	0,467
Consumo de bebida alcoólica	0,67 (1,3)	0,26 (0,75)	0,157
IMC (kg/m^2^)	31,16 (5,78)	30 (4,01)	0,387
PAS antes da FMD (mmHg)	146,71 (21,3)	137,7 (22,2)	0,103
PAD antes da FMD (mmHg)	85,33 (14,4)	83,26 (11)	0,545
HbAc1 (mg/dL)	6,52 (1,0)	5,99 (0,9)	0,020
Glicose sanguínea em jejum (mg/dL)	117,37 (43,7)	99,66 (15,2)	0,064
Colesterol total (mg/dL)	186,55 (63,2)	187,26 (47,4)	0,962
HDL - colesterol (mg/dL)	47,49 (11,8)	49,26 (8,6)	0,522
LDL - colesterol (mg/dL)	124,47 (73,5)	116,22 (45,2)	0,623
Triglicerídeos (mg/dL)	144,33 (109,7)	114,57 (45,9)	0,217
Ácido úrico (mg/dL)	5,54 (1,3)	6,04 (2,1)	0,229
Sódio (mg/dL)	140,04 (2,9)	140,62 (5,5)	0,572
Potássio (mg/dL)	4,23 (0,5%)	4,52 (0,4)	0,029
Proteína C reativa (mg/dL)	5,05 (3,9)	3,37 (3,2)	0,040
Consumo de bebida alcoólica	0,67 (1,3)	0,26 (0,7)	0,157
Prática de atividade física	1,69 (2,4)	1,96 (2,7)	0,681

Células média e desvio padrão. Comparações via teste t para amostras independentes. IMC: índice de massa corporal; PAS: pressão arterial sistólica; PAD: pressão arterial diastólica; HAR: hipertensão arterial resistente.

**Figura 4:**
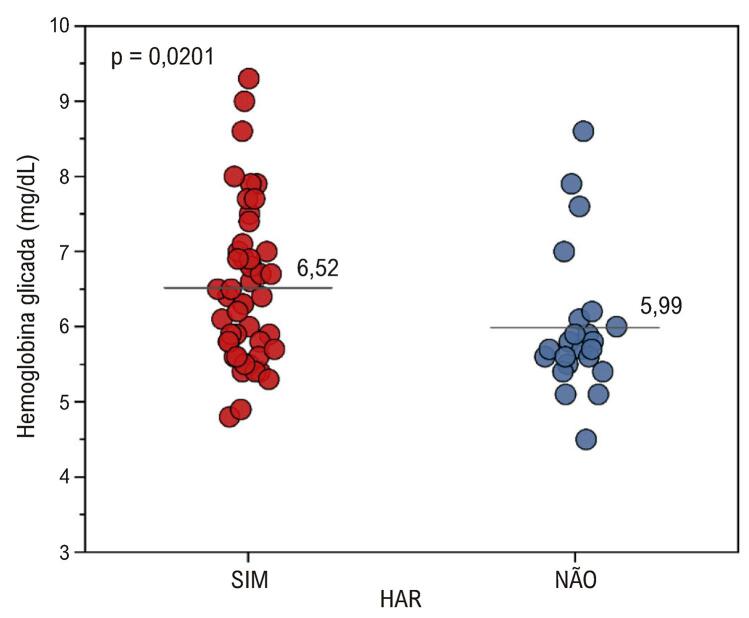
– Comparação de hemoglobina glicada para HAR e Não-HAR. HAR: hipertensão arterial resistente.

**Figura 5:**
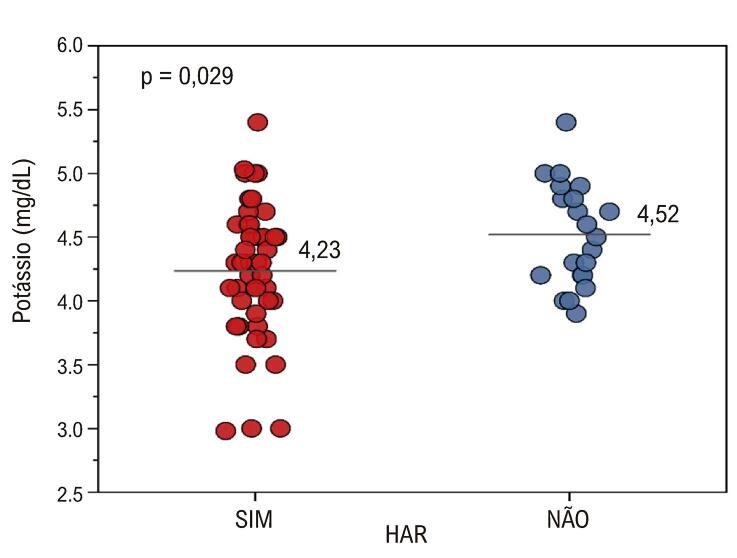
– Comparação em relação ao potássio entre os grupos HAR e não HAR. HAR: hipertensão arterial resistente.

**Figura 6:**
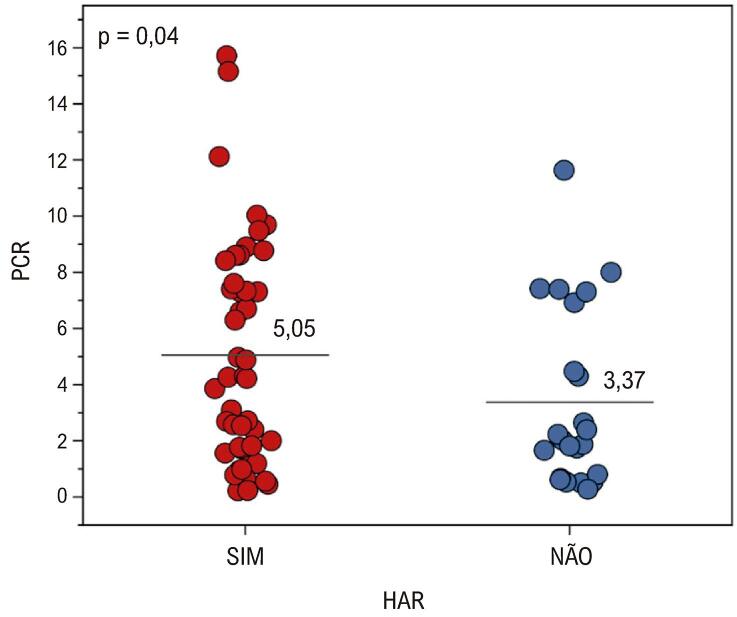
– Comparação em relação à proteína C reativa entre os grupos HAR e não HAR. Foram encontradas diferenças significativas (p = 0,04), e proteína C reativa tem valor médio maior no grupo HAR. HAR: hipertensão arterial resistente; PCR: proteína C reativa. Fonte: autoria própria.

Foram encontradas diferenças significativas em relação à hemoglobina glicada (HbAc1) ( Figura 4 ), ao potássio ( Figura 5 ) e à proteína C reativa (p *=* 0,02; 0,029 e 0,04, respectivamente) ( Figura 6 ). Pacientes com HAR apresentam valores mais elevados de HbAc1 e Proteína C, enquanto o grupo não HAR apresenta valores mais elevados de potássio^.^

Foram encontradas diferenças significativas (p = 0,0201) e a média de hemoglobina glicada é maior no grupo HAR.

Foram encontradas diferenças significativas (p = 0,029) e o potássio tem média maior no grupo HAR.

Foram encontradas diferenças significativas (p = 0,04) e a proteína C reativa tem média maior no grupo HAR.

## Discussão

A disfunção endotelial é uma alteração funcional detectável no processo aterosclerótico e decorre da diminuição da biodisponibilidade do óxido nítrico. Compreender os fatores que causam a disfunção endotelial constitui um desafio constante na pesquisa em saúde, pois a disfunção endotelial pode ser encontrada não apenas em pacientes com doença aterosclerótica clinicamente evidente, mas também em pacientes com fatores de risco. Já a hipertensão arterial é uma doença que atinge indivíduos de diferentes faixas etárias, hábitos de vida e condições de saúde.

O grande desafio dos estudos correlacionais nessas duas áreas é tentar encontrar correlações ou associações significativas entre variáveis contínuas e fatores categóricos que de alguma forma expliquem o risco de desenvolvimento dessas patologias. Existem dois obstáculos sérios aqui: o primeiro é que, na maioria dos casos, não existe um desenho experimental controlado para confirmar as associações. O segundo problema é que a intensidade do tamanho do efeito da associação entre fatores de risco e disfunção endotelial é geralmente baixa, o que dificulta a captura com amostras não muito grandes.

No presente estudo, procuramos correlacionar a FMD e a hipertensão (resistente e não resistente) com vários fatores categóricos e contínuos. Obtivemos muitas associações estatisticamente não significativas (onde seria de se esperar que fossem significativas) e algumas significativas, as quais não eram esperadas, com correlação de sinal oposto, conforme esperado. Explicaremos cada uma delas detalhadamente nos parágrafos seguintes.

**Figura 3:**
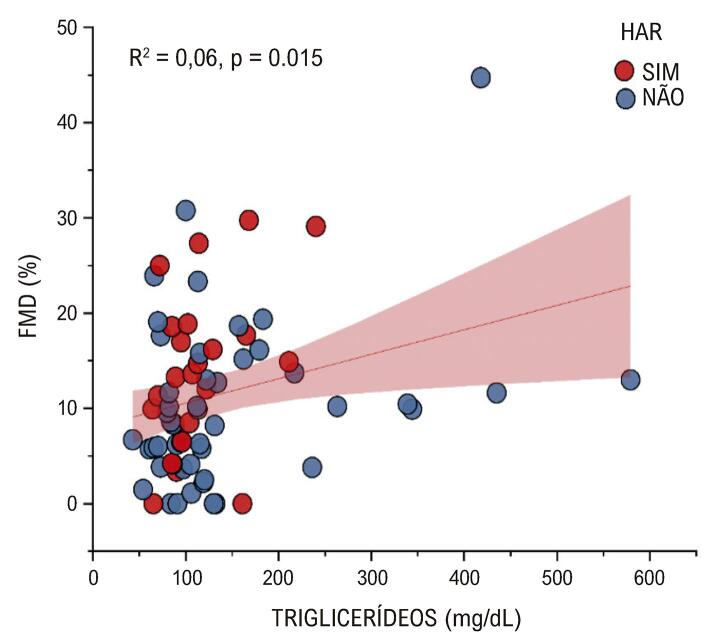
– Regressão linear entre FMD e triglicerídeos. FMD: dilatação mediada por fluxo; HAR: hipertensão arterial resistente.

Vamos começar pela comparação entre as médias de triglicerídeos entre os grupos com DMF alterada e inalterada, que revelou um padrão curioso que seria o oposto do esperado. A média de triglicerídeos foi maior em pacientes sem DMF alterada em comparação com pacientes com DMF alterada, representando um p de significância de 0,015 ( Figura 3 ). Kaplangoray et al.^19^demonstraram que a baixa dilatação está associada a níveis mais elevados de lipídios na corrente sanguínea, assim como nos estudos realizados por Holewijn et al.^20^ e Fernandes.^21^

Sempre que realizamos múltiplos testes estatísticos e encontramos resultados que contradizem um referencial teórico já bem estabelecido na linha de pesquisa, como é o presente caso, é fundamental estar atento aos tamanhos dos efeitos encontrados e ao número de testes estatísticos realizados, empregar alguns critérios de correção que minimizem a chance de erro tipo 1 na interpretação dos valores estatísticos de p e considerar o tamanho da amostra em relação a outros estudos que obtiveram efeitos opostos.^22 - 24^Considerando esses fatores, notamos que, embora existam diferenças significativas, o tamanho do efeito, representado pela estatística t, é muito pequeno, da ordem de 2,31, e o valor de p não é sequer inferior a 0,01, o que não pode ser considerado muito efetivo para descartar a hipótese nula.

Em relação à HAR (resistentes e não resistentes) e às variáveis quantitativas, temos dois resultados significativos nas comparações entre HAR e HbAc1 e entre não HAR e potássio. Quanto ao resultado referente à hemoglobina glicada, aqui novamente temos um resultado que contradiz o que era esperado em teoria. A hemoglobina glicada é maior no grupo HAR do que no grupo não HAR, havendo uma diferença muito pequena entre as médias e o valor de *p* de 0,021, com base em dados de 69 pacientes. Esse resultado contradiz o esperado na literatura. Shimizu et al.,^25^em um estudo conduzido no Japão, encontraram uma relação negativa significativa entre os níveis de HbAc1 e a PA. Ghost et al.^26^descobriram que uma quantidade menor de hemoglobina glicada leva a níveis mais elevados de PA. Portanto, adotamos aqui o rigor do critério de Bonferroni para qualificar a correlação oposta encontrada como não significativa (apenas valores de p inferiores a 0,0031 seriam significativos).

Com relação ao potássio, encontramos resultado significativo, de acordo com as expectativas teóricas, ou seja, quanto maior o nível de potássio, menor a PA, conforme encontrado por Fonseca et al.^27^ Segundo Santos e Vasconcelos,^28^ uma dieta rica em potássio é altamente recomendada para pacientes hipertensos, uma vez que esse nutriente está associado à redução de doenças cardiovasculares e dos níveis pressóricos. Porém, adotando o mesmo rigor de antes, notamos que o tamanho do efeito é pequeno (diferença muito sutil entre as médias, de 0,29). Dado o número de testes estatísticos realizados e o tamanho da amostra de apenas 68 indivíduos para essa comparação, é razoável interpretar este resultado com cautela, embora ele esteja em consonância com o que é previsto pela literatura científica.

No que diz respeito à associação entre medicamentos e HAR e não HAR, encontramos alguns achados significativos condizentes com o esperado na literatura. O primeiro deles é a associação entre pacientes hipertensos resistentes e a variável dos vasodilatadores (17 para 1), em que, por definição, indivíduos com HAR estão mais associados ao uso de vasodilatadores, uma vez que estes são muito úteis no tratamento de várias condições médicas, incluindo hipertensão.^29^

Um desses vasodilatadores, a clonidina, também está mais associado a indivíduos que apresentam hipertensão alterada, o que também é esperado. Como um agonista de função central que diminui o tônus simpático, a clonidina pode ser muito benéfica no tratamento de crises de hipertensão, inclusive em situações pós-operatórias.^30^ Descobrimos também que a espirolactona está mais associada a indivíduos com hipertensão alterada, o que era esperado. Foi demonstrado experimentalmente que a espirolactona reduz consideravelmente as pressões arteriais sistólica e diastólica em pacientes hipertensos.^31^

Um dos desafios para compreender quais fatores afetam a FMD é um ponto de corte definitivo, com suporte teórico científico e estatístico, que defina um valor a partir do qual se possa classificar com segurança se um determinado grupo de pacientes apresenta FMD alterada. Tal classificação ainda não existe na literatura.

Poucos estudos apresentam a tecnologia necessária para determinação da FMD da artéria braquial. Portanto, a dilatação representada pela FMD, embora pareça de fácil implementação, tem demonstrado lacunas quanto aos seus resultados reais, inclusive alterações posturais sutis, inclinação do transdutor, medidas em sístole ou diástole, variação no local de medida, qualidade da imagem obtida e, por fim, o diâmetro da artéria braquial. Artérias com diâmetro inferior a 3,0 mm são mais propensas a erros, pois uma variação de 5% estaria próxima do limite de detecção. Além disso, o horário de realização do exame, o uso de cafeína, o jejum inadequado, o ambiente de prova barulhento ou desconfortável por parte do paciente e a expertise do examinador podem alterar o exame.

No presente estudo, os valores da FMD da artéria braquial direita não eram muito semelhantes à realidade endovascular do perfil da amostra. Um ponto a ser considerado foi o jejum de quatro horas, além da relevância acerca do entendimento de que o rigor da técnica, associado às tecnologias que captam o diâmetro real do vaso, é fundamental para o aprimoramento e o ajuste da metodologia utilizada. Essas medidas podem aprimorar estudos futuros e, assim, consolidar a técnica como ferramenta prática na prevenção de riscos cardiovasculares.

## Conclusões

A função endotelial prejudicada em pacientes hipertensos funciona como um preditor para algumas variáveis clínicas dos pacientes e está correlacionada, embora em intensidade baixa, com o LDL e os triglicerídeos. Os grupos com FMD alterada e não alterada diferem somente em relação aos triglicerídeos. Já os grupos de hipertensos (HAR e não HAR) se diferenciam em relação ao potássio, à proteína C reativa e à hemoglobina glicada. O estudo tem a limitação de não ser um ensaio experimental e também de não possuir uma amostra elevada. Efeitos de intensidade baixa são melhor capturados por tamanhos amostrais elevados. No presente estudo, tivemos menos de 40 pacientes em cada grupo, ainda assim, conseguimos capturar alguns efeitos.Esses achados sugerem que estudos futuros com maior amostragem podem revelar mais correlações interessantes, preditas em teoria.
