# Supplementary material for: Função Endotelial por Dilatação Mediada por Fluxo (FMD) na Artéria Braquial em Hipertensos
Source: Arq Bras Cardiol. 2025 May 15;122(5):e20240533. [Article in Portuguese] doi: 10.36660/abc.20240533 (PMC12108117; doi:10.36660/abc.20240533)
Supplement: Translation

# Translation: Endothelial Function by Flow-Mediated Dilation (FMD) in the Brachial Artery in Hypertensive Patients

**DOI:** 10.36660/abc.20240533i

**Authors:** Elaine Alves Santos Tessier, Carla Daltro, Eduardo Martins Netto, Glauco Moniz de Aragão Doria, Andrea Jimena Gutierrez Peredo, Fabio Bulhões, Roque Aras, Ryan A. Harris

**Affiliations:** 1 Brazil Universidade Federal da Bahia, Salvador, BA – Brazil; 2 USA Augusta University - Laboratory of Integraative and Vascular Exercise Physiology – LIVEP, Augusta, Georgia – USA

**Keywords:** Brachial Artery, Vascular Endothelium, Hypertension

## Abstract

**Background:**

High blood pressure (BP) values have traditionally been associated with the risk of ischemic heart disease, stroke, chronic kidney disease, and early mortality. The brachial artery FMD after cuff deflation has become the standard parameter for quantifying endothelial function, being a useful surrogate outcome due to its non-invasiveness, close correlation with coronary endothelial function, and association with the incidence of long-term coronary events.

**Objectives:**

To test hypotheses of correlation between the FMD and several blood parameters and to compare parameters between altered and non-altered FMD groups, and between hypertensive patients in the resistant hypertension groups (RHTN and non-RHTN).

**Methods:**

Seventy-two volunteers from a referral hypertension outpatient clinic participated in this prospective cross-sectional study, in which several patient variables were compared between the altered FMD (n = 38) and non-altered FMD (n = 34) groups, and also between the RHTN (n = 49) and non-RHTN (n = 23) groups. The variables that would explain the FMD were also investigated in this study. Statistical analyses were performed using parametric methods when the assumptions were met, and non-parametric methods otherwise. The significance level adopted in the statistical analysis was 5%.

**Results:**

The results showed a significant positive correlation between the FMD and LDL (p = 0.204, p = 0.042) and between FMD and triglycerides (p = 0.247, p = 0.037). Glycated hemoglobin was higher in the RHTN group (p = 0.020), potassium was higher in the non-RHTN group (p = 0.029), and C-reactive protein was higher in the RHTN group (p = 0.04). For the other comparisons, no statistically significant differences were found.

**Conclusion:**

LDL and triglycerides are FMD predictors, and the RHTN and non-RHTN groups differ in terms of the amount of potassium, protein C, and glycated hemoglobin. The altered and non-altered FMD groups differ only in terms of triglycerides.

## Introduction

High blood pressure (BP) values have traditionally been associated with the risk of ischemic heart disease, stroke, chronic kidney disease, and early mortality.^1^Resistant hypertension (RHTN) is defined as office BP, remaining ≥ 140/90 mmHg despite treatment with three or more antihypertensive drugs that have synergistic actions at the maximum recommended or tolerated doses. At least one of these drugs should preferably be a thiazide diuretic. When BP control is achieved with four or more antihypertensive drugs, the patient is classified as resistant but controlled hypertensive (BP < 140/90 mmHg).^1 - 4^In Brazil, the multicenter ReHOT (Resistant Hypertension Optimal Treatment) study found a prevalence of 11.7% for this comorbidity.^5^

The primary function of the arterial system is to “efficiently distribute blood to peripheral organs and maintain vascular homeostasis”.^3 , 6^Endothelial dysfunction is the first functional alteration detectable in the atherosclerotic process.^7^This is due to a decreased bioavailability of nitric oxide found not only in patients with clinically evident atherosclerotic disease, but also in patients with risk factors.^8^It is an early manifestation of both atherosclerotic disease and type 2 diabetes mellitus (DM2), and its attenuation may occur soon after the initiation of therapies with antiatherosclerotic effects.^2 , 4^

In 1992, Celejamer et al.^9^developed the flow-mediated dilation (FMD), a noninvasive method to assess early changes in vascular function in systemic arteries. The brachial artery FMD after cuff deflation has become the standard parameter for quantifying endothelial function, being a useful surrogate outcome due to its non-invasiveness, close correlation with coronary endothelial function, and association with the incidence of long-term coronary events.^7^Numerous factors can potentially confound the measurement of FMD.^10^Besides, there is no consensus on aspects such as the location and time of cuff compression, which alters the quantitative result of the examination.^11^

The objective of this study was to test hypotheses of correlation between the FMD and several blood parameters and to compare parameters between groups with altered and non-altered FMD, and between hypertensive patients in the resistant hypertension groups (RHTN and non-RHTN).

## Methods

### Data collection

Seventy-two volunteers from an Arterial Hypertension referral outpatient clinic were included in this prospective cross-sectional study. The patients were informed about the preparation for the examination in advance.

The clinical and demographic characteristics of the patients were collected from electronic medical records. The local ethics committee approved the study (CAE # 81701717.6.0000.0049 and Opinion # 2.635.984), and written informed consent was obtained from all patients before the examinations.

### Right brachial artery flow-mediated dilation (FMD) examination

During the examination, vasodilation in the brachial artery is assessed and occurs in response to the significantly increased blood flow, as induced by a period of circulatory occlusion. Reactive hyperemia is induced by the rapid release of a pneumatic pressure cuff placed around the forearm and inflated to suprasystolic pressure for five minutes. This procedure increases shear stress along the vessel in a parallel and laminar manner, activating mechanoreceptors in endothelial cells and promoting NO release.^12 , 14^

The pressure exerted on the arm causes vascular ischemia and subsequent dilation of the vessels.^15^After five minutes, the deflation control valve is slowly opened. Once it reaches zero on the device, the posterior diameter (D2) is measured. Another 60 seconds are then allowed before applying the formula (D2 – D1)/D1 × 100, where values of 10% or less indicate alterations, as established by Regattieri et al.^16^This calculation provides the values for determining FMD, which is the focus of this study.

All exams were conducted by the same volunteer physician, a specialist in ultrasound for 20 years. During the procedure, patients were also offered the opportunity to undergo carotid and vertebral Doppler (c/v), following the methodology outlined in the 2017 Brazilian Guideline for Dyslipidemia.^17^These exams were always performed before measuring the FMD. The laboratory markers used for the study were: total cholesterol, HDL-cholesterol, LDL-cholesterol, glycated hemoglobin (HbAc1), uric acid, and C-reactive protein (CRP), as collected from electronic medical records.^18^

### Statistical analysis

Data were compiled and analyzed using SPSS^®^software (version 25.0, Chicago, IL Statistical Package for the Social Sciences). In all tests, an alpha significance level of 5% was applied. Categorical variables were expressed as frequency and percentage, and continuous variables were expressed as mean and standard deviation. The chi-square test was used to test associations between categorical factors. When comparing the correlation pattern between quantitative and ordinal variables, the Spearman’s correlation analysis was used, as it better captures positive or negative correlation patterns without the need for perfect linearity. When comparing continuous variables between the RHTN (resistant and non-resistant) and FMD (altered and unaltered) groups, the t-test for independent samples was used. In all tests, normality was tested by the Shapiro Wilk test and homogeneity of variances was tested by the Levene test. Data did not deviate from normality and showed homogeneous variances in all comparisons. Linear regression analyses were also performed between LDL and FMD, and between triglycerides and FMD, with the assumptions of homoscedasticity and normality of the residues tested.

## Results

For objective representation, the Central Illustration illustrates the data in a clear and practical manner.

Initially, several variables between the altered and unaltered FMD groups were compared ( Table 1 ). Significant differences can be noted for triglycerides (p = 0.023), which was higher in the unaltered FMD group ( Figure 1 ).

**Table 1:** – Sociodemographic and clinical profile of hypertensive patients undergoing Flow Mediated Dilation (FMD) in the right brachial artery

Variables	Altered FMD (n = 38)	Unaltered FMD (n = 34)	p
Age (years)	59.13 (10.8)	58.24 (13.02)	0.750
BMI (kg/m^2^)	30.9 (5.56)	30.67 (5.03)	0.855
SBP before FMD (mmHg)	143.39 (20.78)	144.32 (23.32)	0.859
DBP before FMD (mmHg)	85 (14.34)	84.29 (12.4)	0.824
Fasting blood glucose (mg/dL)	109.24 (31.53)	114.24 (43.81)	0.581
HbA1c (mg/dL)	6.31 (1.01)	6.39 (1.05)	0.728
Total cholesterol (mg/dL)	188.37 (64.28)	185 (51.73)	0.809
Triglycerides (mg/dL)	110.51 (65.48)	161 (113.62)	0.023
HDL – cholesterol (mg/dL)	49.01 (12.42)	47.03 (8.84)	0.445
LDL – cholesterol (mg/dL)	116.43 (60.25)	127.63 (71.13)	0.475
Sodium (mg/dL)	139.74 (4.23)	140.78 (3.41)	0.265
Potassium (mg/dL)	4.34 (0.5)	4.29 (0.52)	0.667
Uric acid (mg/dL)	5.69 (1.49)	5.71 (1.76)	0.949
C-reactive protein (mg/dL)	4.87 (4.06)	4.1 (3.43)	0.400
Frequency of alcohol consumption	0.55 (1.06)	0.53 (1.26)	0.932
Exercise frequency	1.53 (2.19)	2.06 (2.82)	0.371

Source: author’s own work. In the mean and standard deviation cells, statistical p was obtained via t-test for independent samples. FMD: flow-mediated dilation; BMI: body mass index; SBP: systolic blood pressure; DBP: diastolic blood pressure.

**Figure 1:** – T-test comparing the amount of triglycerides between the altered and non-altered FMD groups.

When comparing the blood pressure groups, a higher median value was found for the non-RHTN group (43.9) compared to the RHTN group (33), p = 0.039. In addition, the correlations between FMD and all quantitative variables of the study were tested ( Table 2 ).

**Table 2:** – Spearman correlation between FMD and the other quantitative variables

Variables	N	Spearman (Rho)	p
Age	72	-0.004	0.972
BMI	72	0.023	0.844
SBP before FMD	72	-0.005	0.969
DBP before FMD	72	-0.062	0.602
Fasting blood glucose	71	0.015	0.901
HbAc1	71	0.024	0.845
Total cholesterol	72	0.122	0.306
HDL - Cholesterol	71	0.034	0.776
LDL - Cholesterol	71	0.204	0.042
Triglycerides	71	0.247	0.037
Sodium	70	-0.005	0.965
Potassium	70	0.022	0.859
Uric acid	69	-0.028	0.820
C-reactive protein	70	-0.111	0.360
Alcohol consumption	72	-0.100	0.402
Exercise	72	0.106	0.374

FMD: flow-mediated dilation; BMI: body mass index; SBP: systolic blood pressure; DBP: diastolic blood pressure.

When analyzing the descriptive statistics of our sample, the maximum value obtained in the altered FMD group was 11.32, with a mean of 5.25, standard deviation of 3.43 and the minimum value was zero. Unaltered FMD presents a much greater variation (mean of 18.2 and standard deviation of 7.43), with a minimum value of 10.14 and a maximum value of 44.72 ( Table 3 ). When taking the maximum FMD group value as a cutoff point, this value would be 11.32. However, studies designed specifically for this issue of the cutoff point are recommended to further clarify this issue.

**Table 3:** – Maximum and minimum FMD values of the right brachial artery in hypertensive patients

Altered FMD	N	Minimum	Maximum	Mean	Standard deviation
Yes	38	0	11.32	5.25	3.43
No	34	10.14	44.72	18.2	7.43

FMD: flow-mediated dilation.

A significant positive correlation between FMD and LDL and between FMD and triglycerides was found. Regression analyses were conducted for the two significant relationships above (Figures 1 and 2).

The higher the LDL values, the higher the FMD values. LDL correctly predicts and explains 0.8% of the variation in FMD ( Figure 2 ).

**Figure 2:** – Linear regression between FMD and LDL. FMD: flow-mediated dilation; RHTN: resistant hypertension.

Table 4 shows the association between the categorical factors and the RHTN and non-RHTN groups.

**Table 4:** – Resistant and non-resistant hypertensive patients and categorical variables

	Category	RHTN	non-RHTN	p
Altered FMD	Yes	29	9	
	No	20	14	0.112
Smoking	Yes	2	1	
	No	47	22	0.958
Alcohol consumption	Yes	2	0	
	No	35	20	0.299
Exercise	Yes	21	9	
	No	28	14	0.765
BMI	Eutrophic	6	5	
	Overweight	16	8	
	Obese	27	10	0.508
Dysglycemic	Yes	29	14	
	No	20	9	0.892
Vasodilator	Yes	17	1	
	No	32	22	0.006
Atheromatosis	Yes	36	16	
	No	13	7	0.73
	No	13	12	0.033
Statin	Yes	38	16	
	No	11	7	0.466

Associations inferred via chi-square test. FMD: flow-mediated dilation; BMI: body mass index; RHTN: resistant hypertension.

The use of vasodilators is more associated with RHTN patients (0.006). The comparison of the LDL means between the groups that use and do not use statins showed no significant differences (p = 0.336). Table 5 presents comparisons of several parameters between the RHTN and non-RHTN groups.

**Table 5:** – Resistant and non-resistant hypertensives x quantitative variables

Variable	RHTN	non-RHTN	p
Age	59.41 (11.4)	57.22 (12.7)	0.467
Alcohol consumption	0.67 (1.3)	0.26 (0.75)	0.157
BMI (kg/m^2^)	31.16 (5.78)	30 (4.01)	0.387
SBP before FMD (mmHg)	146.71 (21.3)	137.7 (22.2)	0.103
DBP before FMD (mmHg)	85.33 (14.4)	83.26 (11)	0.545
HbAc1 (mg/dL)	6.52 (1.0)	5.99 (0.9)	0.020
Fasting blood glucose (mg/dL)	117.37 (43.7)	99.66 (15.2)	0.064
Total cholesterol (mg/dL)	186.55 (63.2)	187.26 (47.4)	0.962
HDL - cholesterol (mg/dL)	47.49 (11.8)	49.26 (8.6)	0.522
LDL - cholesterol (mg/dL)	124.47 (73.5)	116.22 (45.2)	0.623
Triglycerides (mg/dL)	144.33 (109.7)	114.57 (45.9)	0.217
Uric acid (mg/dL)	5.54 (1.3)	6.04 (2.1)	0.229
Sodium (mg/dL)	140.04 (2.9)	140.62 (5.5)	0.572
Potassium (mg/dL)	4.23 (0.5%)	4.52 (0.4)	0.029
C-reactive protein (mg/dL)	5.05 (3.9)	3.37 (3.2)	0.040
Alcohol consumption	0.67 (1.3)	0.26 (0.7)	0.157
Exercise	1.69 (2.4)	1.96 (2.7)	0.681

Mean and standard deviation cells. Comparisons via t-test for independent samples. BMI: body mass index; SBP: systolic blood pressure; DBP: diastolic blood pressure; RHTN: resistant hypertension.

Significant differences were found for glycated hemoglobin (HbAc1) ( Figure 4 ), potassium ( Figure 5 ), and C-reactive protein (p = 0.02; 0.029 and 0.04, respectively) ( Figure 6 ). Patients with RHTN have higher HbAc1 and Protein C values, while the non-RHTN group has higher potassium values.

**Figure 4:** – Comparison of glycated hemoglobin for RHTN and non-RHTN. RHTN: resistant hypertension.

**Figure 5:** – Comparison for potassium between RHTN and non-RHTN groups. RHTN: resistant hypertension.

**Figure 6:** – Comparison for C-reactive protein between RHTN and non-RHTN groups. Significant differences were found (p = 0.04), and C-reactive protein has a higher mean value in the RHTN group. RHTN: resistant hypertension; CRP: C-reactive protein. Source: Authors.

Significant differences were found (p = 0.0201), and the glycated hemoglobin mean value is higher in the RHTN group.

Significant differences were found (p = 0.029), and potassium has a higher mean value in the RHTN group.

Significant differences were found (p = 0.04), and C-reactive protein has a higher mean value in the RHTN group.

## Discussion

Endothelial dysfunction is a functional alteration observed in the atherosclerotic process, resulting from reduced bioavailability of nitric oxide. Identifying the factors that contribute to endothelial dysfunction remains a challenge in health research. This condition is not only found in patients with clinically evident atherosclerotic disease but also in those with risk factors. High blood pressure affects individuals of various ages, lifestyles, and health conditions.

A key challenge in correlational studies between these two areas is identifying significant associations between continuous variables and categorical factors that help explain the risk of developing these conditions. Two major obstacles arise: first, the absence of a controlled experimental design in most cases, making it difficult to confirm associations; second, the typically low effect size of the association between risk factors and endothelial dysfunction, which complicates detection in small samples.

This study aimed to correlate FMD and hypertension (both resistant and non-resistant) with various categorical and continuous factors. Many expected associations were statistically non-significant (where one would expect them to be significant), while some unexpected correlations were significant, occasionally with an opposite sign. Each will be explained in detail in the following paragraphs.

The mean triglyceride levels were compared between altered and unaltered FMD groups, revealing a surprising pattern opposite to expectations. Mean triglycerides were higher in patients without altered FMD compared to those with altered FMD, representing a significance p of 0.015 ( Figure 3 ). Kaplangoray et al.^19^demonstrated that low dilation is associated with higher levels of lipids in the bloodstream, just as did the studies by Holewijn et al.^20^and Fernandes.^21^

**Figure 3:** – Linear regression between FMD and triglycerides. FMD: flow-mediated dilation; RHTN: resistant hypertension.

When multiple statistical tests yield results that contradict well-established theoretical frameworks in the line of research, as is the case here, it is crucial to consider effect sizes and the number of statistical tests performed adopt correction criteria that minimize the risk of type 1 errors in interpreting *p* -values, and consider the sample size in relation to studies with opposite findings.^22 - 24^Considering these factors, despite the observed significant differences, the effect size—represented by the t statistic—was quite small (approximately 2.31), and the p-value was not even below 0.01. This weakens its reliability for rejecting the null hypothesis.

Regarding resistant and non-resistant RHTN and quantitative variables, two significant results were found in the comparisons between RHTN and HbAc1 ( Figure 4 ) and between non-RHTN and potassium ( Figure 5 ). As for the result of glycated hemoglobin, again the result contradicts what was theoretically expected. Glycated hemoglobin is higher in the RHTN group than in the non-RHTN group, with a very small difference between the mean values and the *p-* value of 0.021, based on data from 69 patients. This result contradicts the literature. Shimizu et al.^25^in a study performed in Japan, found a significant negative relationship between HbAc1 levels and blood pressure, and Ghost et al.^26^found that a lower amount of glycated hemoglobin leads to higher blood pressure levels. Therefore, Bonferroni’s rigorous criterion was used to qualify such opposite correlation found as non-significant (only p-values lower than 0.0031 would be significant).

For potassium, a significant result was found, in accordance with theoretical expectations, that is, the higher the potassium level, the lower the blood pressure, as found by Fonseca et al.^27^According to Santos & Vasconcelos,^28^a diet rich in potassium is highly recommended for hypertensive patients, since this nutrient is associated with a reduction in cardiovascular diseases and blood pressure levels. Applying the same level of scrutiny, the effect size remained small, with only a subtle difference between means (0.29). Given the number of statistical tests performed and the limited sample size of just 68 individuals for this comparison, these results should be interpreted with caution, even though they align with scientific literature predictions.

Regarding the association between medications and RHTN and non-RHTN, significant associations consistent with the literature were found. The first is the association between resistant hypertensive patients and the vasodilator variable (17 to 1), where, by definition, people with RHTN are more associated with the use of vasodilators, as they are very useful in the treatment of several medical conditions, including hypertension.^29^

One of such vasodilators, clonidine, was more frequently associated with altered hypertension, as expected. As a central agonist that reduces sympathetic tone, clonidine can be highly effective in managing hypertensive crises, including postoperative situations.^30^Spironolactone was also more frequently associated with altered hypertension individuals, which was anticipated. Experimental evidence has demonstrated that spironolactone significantly reduces both systolic and diastolic blood pressure in hypertensive patients.^31^

One of the challenges in understanding which factors affect FMD is a definitive cutoff point, with theoretical scientific and statistical support, which defines a value from which it is possible to safely classify whether a given group of patients has altered FMD. This classification does not yet exist in the literature.

Few studies present this technology for determining brachial artery FMD. Therefore, the dilation represented by FMD, although seemingly easy to implement, has shown gaps in its real-world results. Such gaps include subtle postural changes, transducer tilt, measurements in systole or diastole, variations in the measurement site, quality of the image, and the diameter of the brachial artery. Arteries with less than 3.0 mm are more prone to errors, since a variation of 5% would be close to the detection limit. In addition, several external factors can influence the results of the exam, including exam duration, caffeine intake, inadequate fasting, environmental noise, patient discomfort, and the examiner’s expertise.

In this study, the FMD values of the right brachial artery presented values that were not very similar to the endovascular reality of the sample profile. A key consideration was the four-hour fasting period. Additionally, ensuring rigorous technique and employing technologies that accurately measure vessel diameter are essential for refining and standardizing the methodology. These improvements could enhance future studies and establish the technique as a practical tool for cardiovascular risk prevention.

## Conclusion

Impaired endothelial function in hypertensive patients serves as a predictor for certain clinical variables and shows a weak correlation with LDL and triglycerides. The altered and non-altered FMD groups differ only in terms of triglycerides. The hypertensive groups (RHTN and non-RHTN) differ in terms of potassium, C-reactive protein, and glycated hemoglobin. The study has the limitation of not being an experimental study and also of lacking a large sample. Low-intensity effects are more reliably detected in large sample sizes. This study included fewer than 40 patients per group, yet, some effects were still observed. These findings suggest that future studies with larger samples may uncover stronger correlations, as theoretically predicted.
